# Inconsistent medication recommendations for immune-mediated inflammatory diseases across pregnancy, lactation, and paternal preconception: a guideline-based review

**DOI:** 10.3389/fphar.2026.1744957

**Published:** 2026-02-25

**Authors:** Elise Van den Broeck, Sien Lenie, Marc Ferrante, Ellen De Langhe, Barbara Neerinckx, Tom Hillary, Kristel Van Calsteren, Anne Smits, Laure Sillis, Mette Julsgaard, Veerle Foulon, Michael Ceulemans

**Affiliations:** 1 Department of Pharmaceutical and Pharmacological Sciences, KU Leuven, Leuven, Belgium; 2 Department of Gastroenterology, University Hospitals Leuven, Leuven, Belgium; 3 Department of Chronic Diseases and Metabolism, KU Leuven, Leuven, Belgium; 4 Department of Rheumatology, University Hospitals Leuven, Leuven, Belgium; 5 Department of Development and Regeneration, KU Leuven, Leuven, Belgium; 6 Department of Dermatology, University Hospitals Leuven, Leuven, Belgium; 7 Department of Microbiology, Immunology and Transplantation, KU Leuven, Leuven, Belgium; 8 Department of Gynecology and Obstetrics, University Hospitals Leuven, KU Leuven, Leuven, Belgium; 9 Neonatal Intensive Care Unit, University Hospitals Leuven, Leuven, Belgium; 10 L-C&Y, KU Leuven Child & Youth Institute, Leuven, Belgium; 11 Research Foundation Flanders (FWO), Brussels, Belgium; 12 Department of Hepatology and Gastroenterology, Aarhus University Hospital, Aarhus, Denmark; 13 Department of Clinical Medicine, Aarhus University, Aarhus, Denmark; 14 Center for Molecular Prediction of Inflammatory Bowel Disease (PREDICT), Department of Clinical Medicine, Aalborg University, Copenhagen, Denmark

**Keywords:** clinical guidelines, dermatology, gastroenterology, lactation, medication safety, paternal exposure, pregnancy, rheumatology

## Abstract

**Background:**

Immune-mediated inflammatory diseases (IMIDs) often affect women of childbearing age. Since active inflammatory disease is related with impaired reproductive outcomes, effective disease control is crucial. Clinical guidelines aim to support evidence-based shared decision-making. However, inconsistencies between guidelines across specialties may cause confusion, undermine adherence and lead to adverse health outcomes. This study aimed to assess consistency within and across perinatal IMID guidelines in gastroenterology, rheumatology and dermatology regarding medication use during pregnancy, lactation and among prospective fathers, and to identify medications with insufficient safety data.

**Methods:**

A review was conducted in August 2025 using most recent versions of leading international guidelines (N = 11), published between 2015 and 2025. Guideline recommendations for IMID medications were categorized by two independent authors. Data-analysis examined the prevalence and type of consistency, trimester-specific consistency, most common recommendation per medication and the agreement on preconception discontinuation timing.

**Results:**

Within medical specialties, guideline consistency for individual medications was highest for gastroenterology (61/74, 82.4%), followed by rheumatology (165/220, 75.0%) and dermatology (57/94, 60.6%). Across specialties, agreement was greatest between gastroenterology and rheumatology (pregnancy 86.2%, lactation 81.2%, paternal 88.5%) and lowest between gastroenterology and dermatology (pregnancy 51.3%, lactation 40.6%, paternal 47.1%). Trimester-specific recommendations were consistent across guidelines in 33.3% (2/6) of the cases. Agreement on preconception discontinuation timing was 41.7% (5/12) for maternal cases and 66.7% (2/3) for paternal exposure. At least one inconsistency was found in 75.0% of within-guideline and 87.5% of across-guideline comparisons. At medication-class level, most inconsistencies stemmed from insufficient safety data reporting (59.5%), particularly for small molecules (28.6%), immunomodulators (26.1%) and interleukin inhibitors (34.1%).

**Conclusion:**

Inconsistencies between guidelines, both within and across specialties, were frequent and pose challenges for reproductive decision-making in IMID patients. The lack of safety data in reproductive contexts contributed to guideline inconsistencies and revealed the need to prioritize future research on recently marketed pharmacotherapeutic classes, such as small molecules (JAK-inhibitors, PDE4-modulators) and interleukin inhibitors. In the future, guidelines should be updated regularly, include explicit recommendations, integrate reproductive contexts, and adopt unambiguous language, standardized categories and uniform methodological frameworks.

## 1 Introduction

Immune-mediated inflammatory diseases (IMIDs) are chronic conditions characterized by dysregulation of the immune system, resulting in persistent inflammation in specific organs or tissues (McInnes and Gravallese, 2021). These diseases frequently affect people of reproductive age, posing unique challenges in the context of family planning, pregnancy and lactation (Prentice et al., 2025a; Barenbrug et al., 2025; Chen et al., 2023). Effective disease management through preconception counseling is essential for women wishing to conceive (Barenbrug et al., 2025; De Dycker et al., 2025). Ideally, conception should occur during periods of disease remission, as active disease has been associated with adverse maternal, obstetric and neonatal outcomes (e.g., preeclampsia, pregnancy-related hypertension, stillbirth, preterm birth, small for gestational age and admission to the neonatal intensive care unit) (Prentice et al., 2025a; De Dycker et al., 2025; Ewig et al., 2025; Huang et al., 2021).

Pregnancy induces immunological and physiological changes that can alter disease activity, potentially resulting in alleviation or exacerbation of symptoms depending on the underlying condition (Fajardo et al., 2025; Izquierdo et al., 2023; Hazes et al., 2011). To maintain disease control throughout pregnancy, continuation of IMID therapy may be required. However, safety data on medication use during pregnancy and lactation generally remain limited due to the exclusion of pregnant women from clinical trials and the slow pace of post-marketing surveillance (Roque et al., 2022; Sillis et al., 2022; Caritis and Venkataramanan, 2021; Ghalandari et al., 2020). During lactation, the use of medication is a critical factor influencing breastfeeding decisions, with evidence indicating that women requiring maintenance immunosuppressant therapy are less likely to initiate or continue breastfeeding (Whaites et al., 2025; Pilgrim et al., 2025). More recently, increasing attention has been directed to the impact of IMIDs and their treatment on paternal reproductive health. Such conditions have shown to impair spermatogenesis and pregnancy outcomes (e.g., increased risk of early pregnancy loss), especially during active disease (Perez-Garcia et al., 2021; Friedman et al., 2025). To maintain disease control, male patients may need to continue their medication during the periconception period, raising safety considerations.

To address patient’s concerns, counselling by healthcare professionals is essential (Ewig et al., 2025). Therefore, clinicians may rely on clinical guidelines to support evidence-based decision-making and promote informed choices (Barenbrug et al., 2025). However, IMIDs frequently co-occur in the same individual (e.g., psoriasis and arthritis), necessitating involvement from multiple specialists across different clinical specialties. If discrepancies arise between specialty-specific guidelines, patients may be confronted with conflicting recommendations, potentially leading to confusion and concern (Mahadevan et al., 2025a). This in turn may reduce medication adherence and adversely affect maternal and neonatal health outcomes (Mainbourg et al., 2024).

This study aimed to evaluate the consistency within and across international perinatal guidelines from gastroenterology, rheumatology and dermatology concerning medication use for IMIDs during pregnancy, lactation and paternal exposure prior to conception. In addition, the study aimed to identify medications with insufficient safety data, highlighting the need to prioritize them in future research.

## 2 Methods

### 2.1 Study design

This study presents a review of the most recent international clinical guidelines in gastroenterology, rheumatology and dermatology regarding recommendations on the pharmacological management of IMIDs during pregnancy, lactation and paternal exposure prior to conception. The review focused on IMIDs that are prevalent and clinically relevant in women of reproductive age, including Crohn’s disease and ulcerative colitis (gastroenterology), rheumatoid arthritis, spondylarthritis, systemic lupus erythematosus and psoriasis arthritis (rheumatology) and atopic dermatitis, hidradenitis suppurativa and psoriasis (dermatology). Ethical approval was not necessary for this study.

### 2.2 Data collection and categorization

In August 2025, relevant American, British and European guidelines within the selected IMID domains were identified with input from the clinicians representing the medical specialties. Clinicians indicated the leading international guidelines addressing the pharmacological management of the relevant IMIDs and specified the most recent versions. The selected guidelines were included if they provided recommendations for the perinatal period. Included guidelines are summarized in Table 1.

**TABLE 1:** Overview of the selected guidelines by specialty. Table 1 lists all clinical guidelines included in the review. These guidelines were analyzed for recommendations regarding medication safety across pregnancy, lactation and paternal exposure prior to conception.

Year published	Abbreviation	Detailed description
Gastroenterology		
2025	PIANO	Global Consensus Statement on the Management of Pregnancy in Inflammatory Bowel Disease (Mahadevan et al., 2025b)
2022	ECCO	European Crohn’s and Colitis Organization guideline on sexuality, fertility, pregnancy and lactation (Torres et al., 2023)
2019	AGA	American Gastroenterological Association guideline on Inflammatory Bowel Disease in Pregnancy Clinical Care Pathway (Mahadevan et al., 2019)
Rheumatology		
2024	EULAR	European Alliance of Associations for Rheumatology guideline on the use of antirheumatic drugs in reproduction, pregnancy and lactation (Rüegg et al., 2025)
2023	BSR	British Society for Rheumatology guideline on prescribing drugs in pregnancy and breastfeeding: immunomodulatory anti-rheumatic drugs and corticosteroids (Russell et al., 2023)
2020	ACR	American College of Rheumatology guideline for the Management of Reproductive Health in Rheumatic Musculoskeletal Diseases (Sammaritano et al., 2020)
Dermatology		
2025	EuroGuiDerm dermatitis	EuroGuiDerm guideline on atopic eczema (Wollenberg et al., 2025)
2025	EuroGuiDerm psoriasis	EuroGuiDerm guideline for systemic treatment of psoriasis vulgaris (Nast et al., 2023)
2019 & 2020	AAD-NPF	Joint American Academy of Dermatology-National Psoriasis Foundation guidelines of care for the management of psoriasis with systemic non-biologic therapies (Menter et al., 2019) and with biologics (Menter et al., 2020)
2019	EADV AD	Position paper by the European Task Force on Atopic Dermatitis (Vestergaard et al., 2019)
2015	EADV HS	European S1 guideline for the treatment of hidradenitis suppurativa (Zouboulis et al., 2015)

**FIGURE 1:**
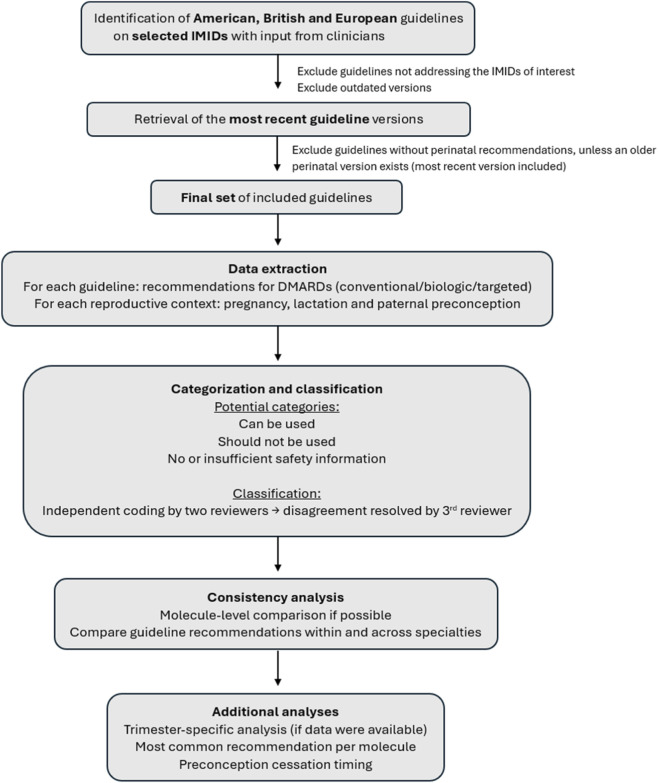
Guideline selection and workflow. Figure 1 provides an overview of the guideline selection process and workflow, including data extraction procedure, categorization and classification of medication recommendations across reproductive contexts, and consistency and additional analyses.

Although the guidelines covered a wide spectrum of medications, only recommendations regarding disease-modifying antirheumatic drugs (DMARDs) – including conventional, biologic and targeted agents–with at least one approved indication in gastroenterology, rheumatology or dermatology were included. For each medication, all relevant guideline information on its use during the three reproductive context (pregnancy, lactation and paternal preconception exposure) was extracted and compiled into an Excel file. That information was then categorized using a simplified three-category model, adapted from Nörby et al. (Nör et al., 2021). The three categories were “can be used” (combining the original categories ‘can be used’, ‘individual benefit-risk assessment’ and ‘trimester-specific information), “should not be used” and “no or insufficient safety information available” (merging the categories ‘not classifiable’ and ‘no available information’). Categorization was performed independently by two authors (E.V.D.B. and S.L.) and discrepancies were resolved by another author (M.C.). The selection procedure and workflow are illustrated in Figure 1.

### 2.3 Data analysis

Descriptive statistics were applied using absolute numbers and percentages. Consistency, both within and across specialties, was defined as the percentage agreement on medication recommendations for molecules mentioned in both guidelines (one-by-one comparison), assessed at molecule level when possible or at medication-class level if needed. Medications that appeared in more than two guidelines were considered multiple times, increasing their weight in the total consistency score. Guidelines without data for a specific reproductive context (e.g., paternal use) were only excluded from the corresponding analysis for that context. Medications with only a single recommendation within a specialty, but with comparable data across specialties, were exclusively included in the cross-specialty analysis. In addition to the consistency analysis, the type of discrepancy and involved medications were qualitatively assessed. For medications with trimester-specific recommendations, a separate analysis was performed to enable a more granular comparison across guidelines. For each medication, the most frequently cited recommendation across guidelines was also identified, along with the number of guidelines supporting it. Finally, recommendations on the timing of maternal and paternal medication discontinuation prior to conception were compared. To ensure a complete overview, information from the Summary of Product Characteristics (SmPC) was incorporated for this latter analysis.

## 3 Results

### 3.1 Comparison of guidelines within each specialty

Comparative analyses of guideline recommendations within each specialty are presented in Table 2. For pregnancy-related recommendations, gastroenterology guidelines demonstrated the highest level of consistency (92.1%), followed by dermatology (78.0%) and rheumatology (68.9%). Consistency levels were generally higher for pregnancy than for lactation or paternal exposure. Within gastroenterology, paternal comparisons were not feasible, as the ECCO guideline was the only one addressing this context. No comparisons could be made between hidradenitis suppurativa and atopic dermatitis due to non-overlapping medications. Between hidradenitis suppurativa and psoriasis, consistency was limited to 50.0% (2/4) for pregnancy-related recommendations and was absent (0%, 0/2) for lactation. Paternal use could not be compared, as the EADV HS guideline did not address this context.

**TABLE 2:** Consistency of guideline recommendations within specialties (%). Table 2 shows the proportion of consistent medication safety recommendations within clinical guidelines in gastroenterology, rheumatology and dermatology for pregnancy, lactation and paternal exposure prior to conception. Consistency reflects the percentage of molecule-specific recommendations aligned between guidelines with available data. Totals are based on all guideline comparisons and may count medications multiple times if they appear in multiple guidelines. Paternal recommendations in gastroenterology were exclusively available in the ECCO guideline, making comparisons within the specialty for this context not feasible. Abbreviations: ECCO (European, gastroenterology), PIANO (global consensus, gastroenterology), AGA (American, gastroenterology), EULAR (European, rheumatology), BSR (British, rheumatology), ACR (American, rheumatology), EuroGuiDerm (European, dermatology), EADV (European, dermatology), AAD (American, dermatology).

Guideline comparisons	Pregnancy	Lactation	Paternal
Gastroenterology guidelines			
ECCO vs. PIANO	16/16 (100.0)	7/12 (58.3)	—
ECCO vs. AGA	10/12 (83.3)	10/14 (71.4)	—
PIANO vs. AGA	9/10 (90.0)	9/10 (90.0)	—
Total	**35/38 (92.1)**	**26/36 (72.2)**	—
Rheumatology guidelines			
EULAR – BSR	20/27 (74.7)	20/28 (71.4)	22/28 (78.6)
EULAR – ACR	18/25 (72.0)	20/24 (83.3)	18/23 (78.3)
BSR – ACR	13/22 (59.1)	20/22 (90.9)	14/21 (66.7)
Total	**51/74 (68.9)**	**60/74 (81.1)**	**54/72 (75.0)**
Dermatology guidelines			
*Atopic dermatitis*			
EuroGuiDerm vs. EADV	5/5 (100.0)	2/4 (50.0)	4/4 (100.0)
*Psoriasis*			
EuroGuiDerm - AAD	12/16 (75.0)	6/6 (100.0)	2/11 (18.2)
*Psoriasis* vs*. atopic dermatitis*			
EuroGuiDerm psoriasis vs. EuroGuiDerm dermatitis	2/2 (100.0)	2/2 (100.0)	0/2 (0.0)
EuroGuiDerm psoriasis vs. EADV dermatitis	3/3 (100.0)	1/3 (33.3)	0/3 (0.0)
AAD vs. EuroGuiDerm dermatitis	3/4 (75.0)	3/3 (100.0)	1/3 (33.3)
AAD vs. EADV dermatitis	3/5 (60.0)	2/4 (100.0)	2/4 (50.0)
Total	**13/16 (81.3)**	**8/13 (61.5)**	**3/13 (23.1)**
*Hidradenitis suppurativa* vs*. psoriasis*			
EADV HS vs. EuroGuiDerm psoriasis	2/2 (100.0)	—	—
EADV HS vs. AAD	0/2 (0.0)	0/2 (0.0)	—
Total	**2/4 (50.0)**	**0/2 (0.0)**	—
*All dermatology guidelines combined*			
Total	**32/41 (78.0)**	**16/25 (64.0)**	**9/28 (32.1)**

The bolt values represent the total number of medications compared across guidelines within a specialty. The denominator indicates the total number of evaluated medications, while the numerator represents the medications with consistent recommendations.

Across all reproductive contexts combined, at least one inconsistency was found in 83.3% of gastroenterology guideline comparisons (5/6), 100% of rheumatology comparisons (9/9) and 61.9% of dermatology comparisons (13/21). Each denominator reflects the total number of guidelines compared within that specialty, while the numerator indicates how many of those comparisons showed at least one inconsistency.

Recommendation inconsistencies, calculated at the medication-class level per specialty and across all three reproductive domains combined, were predominantly driven by insufficient data reporting (37/55) – defined as cases where one guideline cited insufficient safety data while another either permitted (12/37) or contraindicated use (25/37). In gastroenterology, insufficient safety data was the leading cause of inconsistencies (11/12), mostly involving small molecules (5/11). Similarly, in rheumatology, 16 out of 21 inconsistencies were due to insufficient data, also particularly on small molecules (6/16). In dermatology, insufficient data accounted for 10 out of 22 inconsistencies, mainly affecting immunomodulators (4/10) and PDE4-modulators (4/10). Conflicting recommendations – defined as direct contradictions between ‘can be used’ and ‘should not be used’ – were less frequent in gastroenterology (1/12) and rheumatology (5/21). However, in dermatology, most inconsistencies stemmed from conflicting recommendations (12/22), primarily concerning immunomodulators (9/12) (mainly ciclosporin in lactation and methotrexate for paternal exposure). Supplementary Table S1 summarizes guideline inconsistencies by medication class and molecule.

### 3.2 Comparison of guidelines across different specialties

Guideline alignment across the different specialties was highest between gastroenterology and rheumatology for all reproductive contexts, followed by rheumatology and dermatology, and gastroenterology and dermatology (Table 3).

**TABLE 3:** Consistency on medication-specific guideline recommendations across specialty domains (%). Table 3 presents the proportion of consistent recommendations between clinical guidelines across gastroenterology, rheumatology and dermatology across pregnancy, lactation and paternal exposure. Consistency is expressed as the number of aligned recommendations over the total number of compared recommendations.

​	Pregnancy	Lactation	Paternal
Gastroenterology vs. Dermatology			
Combined total	39/76 (51.3)	26/64 (40.6)	8/17 (47.1)
Gastroenterology vs. Rheumatology			
Combined total	94/109 (86.2)	82/101 (81.2)	23/26 (88.5)
Rheumatology vs. Dermatology			
Combined total	67/122 (54.9)	42/94 (44.7)	47/107 (43.9)

Cross-specialty comparisons revealed notable inconsistencies in medication recommendations. The lowest level of consistency was observed in comparisons involving the hidradenitis suppurativa guideline, with 0% consistent medication recommendations. In contrast, a higher level of consistency was observed for pregnancy-related recommendations between gastroenterology and atopic dermatitis (86.7%), and rheumatology and atopic dermatitis (83.3%). Comparisons on paternal medication use were not possible for 20 of the 39 potential guideline comparisons, due to missing or absent data in the corresponding guidelines. A detailed overview of the guideline combinations assessed is provided in Supplementary Table S2.

Across all compared guidelines in pregnancy, lactation and paternal exposure, 84 of 96 guideline comparisons (87.5%) showed at least one inconsistency. When analyzed at the medication-class level per specialty, most inconsistencies stemmed from insufficient safety data reporting (56.6%, 82/145), particularly for interleukin inhibitors (25/82), immunomodulators (20/82, mainly methotrexate in the paternal context [11/20]) and JAK-inhibitors (19/82). Conflicting information accounted for 63 of 145 inconsistencies, mainly involving immunomodulators (37/63) and TNF inhibitors (18/63). These findings are detailed in Supplementary Table S3.

Across guidelines, the most frequently assigned recommendation per medication was analyzed for all 45 included medications. For some medications, no predominant recommendation emerged due to evenly distributed guideline recommendations, particularly for JAK-inhibitors (13.3%). Insufficient safety data was most often reported for interleukin inhibitors (14.8%) and for small molecules (8.9%), mainly JAK-inhibitors (25%) and PDE4-modulator apremilast (9.4%). Details are provided in Supplementary Table S4.

### 3.3 Trimester-specific recommendations

The conventional therapies cyclophosphamide and mycophenolate mofetil showed consistent recommendations across trimesters in the EULAR (2024) (both medications) and ACR (2020) guidelines (cyclophosphamide only, as mycophenolate was not included) (i.e., contraindicated in the first trimester and only considered in cases of severe maternal disease during the second and third trimesters). In the main analysis, the consensus classification for both was ‘should not be used’. In contrast, trimester-specific recommendations for TNF inhibitors adalimumab, etanercept, golimumab and infliximab varied across guidelines. The ACR guideline (2020) permitted use in the first and second trimesters but contraindicated it in the third. Conversely, the EuroGuiDerm psoriasis (2025) contraindicated use in the second and third trimesters and did not specify recommendations for the first trimester. Second-trimester recommendations thus differed across guidelines. In the main analysis, adalimumab, etanercept, golimumab and infliximab were classified as ‘can be used’ in ACR and as ‘should not be used’ in EuroGuiDerm psoriasis, explaining inconsistencies across these guidelines.

### 3.4 Timing of medication cessation prior to conception

Table 4 presents the timing of maternal and paternal preconception medication discontinuation mentioned in the various guidelines and the SmPCs. For women, guideline and SmPC recommendations varied considerably. Methotrexate discontinuation was recommended from 1 to 6 months, with 3 months most frequently advised (4/10). Cyclophosphamide was generally advised to be stopped 3 months prior to conception (2/3), although the SmPC suggested a longer period of 6–12 months. Mycophenolate mofetil was mostly advised to be stopped 6 weeks prior to conception (4/5), except for the EADV guideline which recommended 3 months. In contrast, consistent guidance was found for acitretin (3/3), tofacitinib (2/2), upadacitinib (2/2), ozanimod (2/2) and etrasimod (2/2). Recommendations for JAK-inhibitors (as a group), apremilast and dimethyl fumarate could not be compared due to either insufficient data reporting, missing details on cessation timing (despite being contraindicated) or guidance from only one guideline. For paternal exposure, recommendations were generally consistent across guidelines and in agreement with the SmPC, with the exception of cyclophosphamide. The EULAR and ACR guidelines advised discontinuation at least 3 months prior to conception, whereas the corresponding SmPC recommended 6–12 months.

**TABLE 4:** Guideline-based recommendations for timing of maternal and paternal medication discontinuation prior to conception. Table 4 represents the guideline-based recommendations for medication cessation timing. The Summary of Product Characteristics (SmPC) information was derived from Section 4.6 (Fertility, pregnancy and breastfeeding). The ECCO and EADV HS guidelines did not include information on preconception medication cessation and were not included in the table. Empty cells indicate that the corresponding guideline did not provide a recommendation regarding preconception medication use. Abbreviations: PIANO (global consensus, gastroenterology), AGA (American, gastroenterology), EULAR (European, rheumatology), BSR (British, rheumatology), ACR (American, rheumatology), EGD (EuroGuiDerm: European, dermatology), EADV (European, dermatology), AAD (American, dermatology) SmPC (Summary of Product Characteristics: European, regulatory document).

Medication	Gastroenterology		Rheumatology			Dermatology				​
	PIANO	AGA	EULAR	BSR	ACR	EGD psoriasis	EGD dermatitis	EADV	AAD	SmPC
WOMEN										
Methotrexate	1 month	3 months	1–3 months	1 month	1–3 months	3 months	3 months	6 months	3 months	6 months
Cyclophosphamide	​	​	3 months	​	3 months	​	​	​	​	6–12 months
Mycophenolate mofetil	​	​	6 weeks	6 weeks	6 weeks	​	​	3 months	​	6 weeks
Acitretin	​	​	​	​	​	3 years	​	​	3 years	3 years
JAK-inhibitors (general)	​	​	​	2 weeks	​	​	​	​	​	​
Tofacitinib	4 weeks	​	​	​	​	​	​	​	​	4 weeks
Filgotinib	4 weeks	​	​	​	​	​	​	​	​	1 week
Upadacitinib	4 weeks	​	​	​	​	​	​	​	​	4 weeks
Ozanimod	3 months	​	​	​	​	​	​	​	​	3 months
Etrasimod	1–2 weeks	​	​	​	​	​	​	​	​	2 weeks
Apremilast	​	​	​	​	​	4 weeks	​	​	​	No data
Dimethyl fumarate	​	​	​	​	​	Timing not specified	​	​	​	No data
MEN										
Methotrexate	​	​	​	​	​	​	3 months	3 months	3 months	3 months
Cyclophosphamide	​	​	3 months	Timing not specified	12 weeks	​	​	​	​	6–12 months
Mycophenolate mofetil	​	​	​	​	​	​	​	3 months	​	90 days

## 4 Discussion

### 4.1 Main findings

This study aimed to assess the consistency of perinatal IMID guideline recommendations within and across gastroenterology, rheumatology and dermatology, and to identify medication classes for which perinatal safety data are limited or lacking.

A first key finding is that at least one inconsistency was found in 75.0% of within-specialty comparisons (27/36) and in 87.5% of across-specialty comparisons (84/96). Inconsistencies across specialties in treating different types of IMIDs, with varying degrees of disease-related risks for the mother and (unborn) child and different risk-benefit assessments during the perinatal period, are not entirely unexpected (e.g., the differing impact of a lupus flare compared to mild psoriasis affecting the need for medication). However, three key factors may explain this high rate of inconsistencies: variation in guideline publication dates, insufficient safety data available at the time of guideline publication and evolving regulatory recommendations. First, outdated guidelines contributed substantially to inconsistencies. A striking example is the 2015 EADV guideline on hidradenitis suppurativa (Zouboulis et al., 2015), showing 0% consistency across all comparisons. Although newer versions of this guideline exist, the 2015 guideline was the only one addressing the topic of pregnancy and lactation. The guideline surprisingly contraindicated the use of TNF inhibitors in these contexts, despite extensive safety evidence having been published over the past decade (Ewig et al., 2025; Beltagy et al., 2021; Li et al., 2025; N et al., 2024). This underscores the importance of regularly updating clinical guidelines and ensuring that reproductive contexts are more thoroughly integrated into these revisions (Clark et al., 2006). Second, insufficient safety data often contributed to inconsistent recommendations. Some guidelines explicitly acknowledged the insufficiency of safety data, while others recommended against the use of certain medications without clarifying whether this was due to limited data or documented harm (Duricova et al., 2025). These variations in language, wording or formulation of recommendations introduced ambiguity, especially when recommendations such as ‘should not be used’ were stated without further explanation (see Supplementary Figure S1). This issue was also reflected in our consensus assignment, revealing conflicting recommendations, mainly for small molecules, arising from discrepancies between ‘should not be used’ and ‘insufficient data’ classifications. Notably, the recommendation ‘should not be used’ is stricter and may result in complete avoidance of the medication in clinical practice. Third, changes in regulatory guidance and varying level of adherence to this guidance in clinical guidelines may also have attributed to inconsistencies. This is illustrated by the controversy surrounding paternal safety of mycophenolate prior to conception between 2015 and 2017. The European Medicines Agency (EMA) updated the SmPC of mycophenolate in 2015 to advise contraception for sexually active men during treatment and for 90 days after discontinuation (European Medicines Agency, 2015). This position has been criticized by teratology experts, arguing that the recommendation lacked any scientific evidence (European Netwerk Of Teratology Information Services, 2016). In response, EMA revised its guidance in 2017, softening the language in the SmPC (European Medicines Agency, 2017). These regulatory changes could have introduced variability, or at least some uncertainty, in guidelines.

Three other factors likely contributing to inconsistencies include differences in evidence grading systems across guidelines, vague phrasing of recommendations, and the use of efficacy based rather than safety-based considerations. First, variations in evidence-grading systems used across guidelines may have influenced how available evidence is interpreted and how recommendations are formulated. Supplementary Table S5 provides an overview of the different methodological frameworks and approaches applied in the various guidelines. For example, while the 2022 ECCO guideline ranked evidence by study design (with randomized controlled trials generally lacking for medication use in pregnancy and lactation), the 2025 PIANO Global Consensus guideline used a combined approach: evidence-based when sufficient evidence exists (GRADE) and expert-derived consensus (RAND/UCLA) when evidence was limited, providing (more) expert-driven guidance. Since the various guidelines relied on different methodological frameworks, recommendations may diverge even when based on the same underlying evidence. Second, in certain cases, guideline recommendations lacked explicit classification, leaving substantial room for interpretation. This ambiguity may have contributed to divergent assessments in our study, but even more importantly, may similarly affect interpretation in clinical practice (Codish and Shiffman, 2005). The absence of clear categorization underscores the need for more structured and transparent guideline formulations to reduce confusion and enhance clinical applicability (Codish and Shiffman, 2005). Lastly, some guideline recommendations were not exclusively based on safety aspects but on therapeutic efficacy or the composition of pharmaceutical preparations. For example, ciclosporin was advised against during lactation by the AAD guideline, not because of the active substance itself, but due to the presence of ethanol as an excipient in the preparation. The reason of not recommending the use of a specific medication or product should be clearly emphasized in the guidelines.

A second key finding of our study is that most guideline inconsistencies were linked to insufficient safety data reporting (59.5%), particularly for newer medication classes such as interleukin inhibitors (34.1%) and small molecules (28.6%). This aligns with previous research, highlighting the lack of reproductive safety data for recently approved medications due to the exclusion of pregnant women from clinical trials due to ethical, medicolegal and commercial concerns (Roque et al., 2022; Sillis et al., 2022; Caritis and Venkataramanan, 2021; Ghalandari et al., 2020). Although animal studies raised concerns about malformations with tofacitinib, upadacitinib and ozanimod during pregnancy (Duricova et al., 2025; Monfared et al., 2023), emerging human data and real-world observations have shown more reassuring outcomes during pregnancy and lactation (Duricova et al., 2025; Monfared et al., 2023; Dubinsky et al., 2024; Ernest-Suarez et al., 2024; Clowse et al., 2016; Mitrova et al., 2025; Mahadevan et al., 2024), yet further research is acquired. Similarly, safety data for monoclonal antibodies vedolizumab and ustekinumab remain somewhat limited, though recent studies have suggested favorable safety profiles during pregnancy and lactation (Laube et al., 2021; Prentice et al., 2025b; Chambers et al., 2025). However, most studies mainly focused on short-term maternal and neonatal outcomes, while long-term effects remain largely unknown (Beltagy et al., 2021). For biologics, some studies suggested minimal impact on neonatal immune function (Mitrova et al., 2024; Ernest-Suarez et al., 2025), but for small molecules such as tofacitinib, evidence is extremely limited, with only one study evaluating its effect on the infant’s immune system (Ernest-Suarez et al., 2024). Previous research has shown that it may take up to 27 years before a medication is assigned a specific risk category during pregnancy and lactation (Sillis et al., 2022; Favre et al., 2024). Differences in guideline thresholds for what constitutes as sufficient safety data may have influenced how recommendations were formulated and contributed to guideline inconsistencies. Regarding paternal preconception exposure, safety data are especially scarce, with only a few studies reporting reassuring outcomes on the use of vedolizumab and ustekinumab (Mahadevan et al., 2017; Grosen et al., 2019; Cohen et al., 2020; Grosen et al., 2022; Mahadevan et al., 2022). These findings underscore the need for more research on newer medications and paternal exposure in the reproductive contexts.

The high level of inconsistencies for individual medications across guidelines is concerning, especially given that IMID patients often receive multidisciplinary care (McInnes and Gravallese, 2021) and hence, can receive conflicting advice. This may reduce their medication adherence and adversely affect maternal and neonatal outcomes (Mainbourg et al., 2024). To ensure alignment with current evidence and support evidence-based decision-making in clinical practice, guidelines should be updated regularly and explicitly incorporate reproductive contexts in each version (Clark et al., 2006; Gotfredsen et al., 2025). A major challenge remains the lack of sufficient safety data, which forces clinicians to make treatment decisions without adequate evidence, potentially leading to unnecessary cessation of effective treatment or even termination of pregnancies, and influencing patients’ decisions to remain voluntary childless or to have fewer children (De Dycker et al., 2025; Ghalandari et al., 2020; Laube et al., 2021). A recent international survey, involving 856 gastroenterologists (of whom 61% IBD specialists), identified limited safety data as a key reason for stopping biologic or small molecule therapy during pregnancy and lactation (Casanova et al., 2025). Similarly, conflicting information between guidelines and SmPCs for patients with rheumatic musculoskeletal diseases during pregnancy and lactation frequently led to treatment cessation and suboptimal care (Schreiber et al., 2025). Addressing this evidence gap requires not only more safety research but also more harmonization in how safety data are collected and reported. Recent initiatives have aimed to develop common data models and harmonized reporting to accelerate the generation of robust safety data in this vulnerable population (Favre et al., 2024; Meissner et al., 2021). Standardized reporting is essential to enable pooling across different registries and countries (Richardson et al., 2023; Jiao et al., 2025). Building on these challenges, our study emphasizes the importance of international and interdisciplinary collaborations and highlights the urgent need to expand research on medication safety in reproductive contexts. Future efforts should focus on establishing large-scale, harmonized data registries that include pregnant and lactating women, as well as prospective fathers, who are often overlooked in current research (Georgiou et al., 2025).

### 4.2 Strengths and limitations

Our study offers several strengths. First, its comprehensive design allowed for the inclusion of multiple IMID diseases in reproductive contexts across three major clinical specialties. By applying a robust methodological approach, that included independent categorization of guideline recommendations by two authors, we aimed to minimize subjectivity to ensure consistent and transparent classification, and to enhance reliability of our findings. Second, the inclusion of international guidelines ensured a broad and representative scope of the most relevant and widely used guidelines, increasing the generalizability of our conclusions. Finally, the analysis included paternal preconception exposure, a context that is often overlooked and excluded from clinical research despite the safety questions clinicians and patients may have (Georgiou et al., 2025).

However, the study also has some limitations. First, categorizing recommendations was sometimes challenging due to ambiguous phrasing of the information in the guideline. Some guidelines permitted the use of a medication only as a last resort or did not express a clear preference, which may have led to some oversimplification when assigning categories. In some cases, guidelines provided research data without offering clear communication (e.g., only the number of milk samples analyzed), leaving room for interpretation. Second, trimester-specific recommendations were not considered in the main analysis, however we performed additional analysis taking into account these recommendations. Third, total consistency percentages may not be entirely representative for all included medications due to the presence of certain medications in more or all guidelines, while those included in only one guideline contributed less to the total denominator and percentage. Fourth, our review focused only on conventional, biologic and targeted DMARDs and therefore may lack insight into other frequently used medications among IMID patients (e.g., NSAIDs, colchicine). Finally, an evaluation of medication dosages and neonatal monitoring following prenatal exposure was beyond the scope of this review, as was a formal quantitative assessment of the methodological quality of the included guidelines.

## 5 Conclusion

This study revealed a high level of inconsistencies in clinical guideline recommendations within and across medical specialties concerning the use of medications in patients with immune-mediated diseases during pregnancy, lactation and among fathers prior to conception. These findings highlight the absence of a global harmonized guidance for pharmacological management of IMID diseases in the perinatal period. Contributing factors to these discrepancies include the timing of guideline publication, the absence of explicit and unambiguous recommendations, standardized terminology and/or uniformly applied grading systems across guidelines, as well as the underlying reasons explaining a recommendation. Moreover, the results underscore the limited availability of safety data for many medication classes in the context of pregnancy, lactation, and paternal exposure, particularly for newer therapeutic agents such as interleukin inhibitors, JAK-inhibitors, and PDE4-modulators. In the future, guidelines should be updated regularly, include explicit recommendations, integrate reproductive contexts, and adopt unambiguous language, standardized categories and uniform methodological frameworks to reduce ambiguity.
