# Supplementary material for: Inconsistent medication recommendations for immune-mediated inflammatory diseases across pregnancy, lactation, and paternal preconception: a guideline-based review
Source: Front Pharmacol. 2026 Feb 25;17:1744957. doi: 10.3389/fphar.2026.1744957 (PMC12975931; doi:10.3389/fphar.2026.1744957)
Supplement: Supplementary file 1 [file Table1.docx]

**Supplementary Table 1** provides a detailed overview of the observed inconsistencies within field comparisons, specifying the guidelines involved, the drug class and (if available) the molecule. If only class-level data were available, ‘NA’ was listed in the column ‘Molecule’. **Abbreviations**: ECCO (European, gastroenterology), PIANO (global consensus, gastroenterology), AGA (American, gastroenterology), EULAR (European, rheumatology), BSR (British, rheumatology), ACR (American, rheumatology), EuroGuiDerm dermatitis (European, dermatology), EADV dermatitis (European, dermatology), EuroGuiDerm psoriasis (European, dermatology), AAD (American, dermatology) EADV HS (European, dermatology).

| Supplementary Table 1. Detailed overview of Guideline-Specific Inconsistencies in Medication Recommendations | | | |
| --- | --- | --- | --- |
| **PREGNANCY** | | | |
| **Type of inconsistency** | **Compared guidelines** | **Medication class** | **Molecule** |
| Gastroenterology | | | |
| Insufficient data vs should not be used | AGA vs ECCO | JAK-inhibitor | Tofacitinib |
|  | AGA vs PIANO | JAK-inhibitor | Tofacitinib |
| Can be used vs should be used | ECCO vs AGA | Immunomodulator | Ciclosporin |
| Rheumatology | | | |
| Insufficient data vs should not be used | EULAR vs BSR | Immunomodulator | Leflunomide |
|  |  | JAK-inhibitor | Tofacitinib  Filgotinib  Baricitinib  Upadacitinib |
|  |  | Interleukin inhibitor | Guselkumab  Risankizumab |
|  | EULAR vs ACR | Immunomodulator | Leflunomide |
|  | ACR vs BSR | JAK-inhibitors | Tofacitinib  Baricitinib |
| Can be used vs should not be used | EULAR vs ACR | Interleukin inhibitor | Ustekinumab  Anakinra  Tocilizumab  Secukinumab |
|  |  | Monoclonal antibodies | Belimumab  Abatacept |
|  | BSR vs ACR | Interleukin inhibitor | Ustekinumab  Anakinra  Tocilizumab  Secukinumab |
|  |  | Monocloncal antibodies | Belimumab  Abatacept |
|  | ACR vs BSR | Immunomodulator | Cyclophosphamide |
| Dermatology | | | |
| Insufficient data vs should not be used | AAD vs EuroGuiDerm psoriasis | PDE4-modulator | Apremilast |
|  | AAD vs EADV dermatitis | PDE4-modulator | Apremilast |
| Can be used vs should not be used | AAD EuroGuiDerm psoriasis | TNF-inhibitor | Adalimumab  Infliximab  Etanercept |
|  | EADV dermatitis vs AAD | Immunomodulator | Azathioprine |
|  | AAD vs EADV HS | TNF-inhibitor | Adalimumab  Infliximab |
| **LACTATION** | | | |
| Gastroenterology | | | |
| Insufficient data vs should not be used | ECCO vs PIANO | JAK-inhibitor | Tofacitinib  Filgotinib |
|  |  | S1P-modulator | Ozanimod |
|  | AGA vs ECCO | Immunomodulator | Methotrexate |
|  | AGA vs PIANO | S1P-modulator | Ozanimod |
| Insufficient data vs can be used | ECCO vs PIANO | Anti-integrin | Vedolizumab |
|  |  | Interleukin-inhibitor | Ustekinumab |
|  | ECCO vs AGA | Immunomodulator | Ciclosporin |
|  |  | Anti-integrin | Vedolizumab |
|  |  | Interleukin inhibitor | Ustekinumab |
| Rheumatology | | | |
| Insufficient data vs should not be used | EULAR vs BSR | Immunomodulator | Methotrexate  Leflunomide  Cyclophosphamide  Mycophenolate mofetil |
|  |  | JAK-inhibitor | Tofacitinib  Filgotinib  Baricitinib  Upadacitinib |
|  | EULAR vs ACR | Immunomodulators | Methotrexate  Leflunomide  Cyclophosphamide  Mycophenolate mofetil |
|  | ACR vs BSR | JAK-inhibitors | Tofacitinib  Baricitinib |
| Dermatology | | | |
| Insufficient data vs should not be used | EuroGuiDerm psoriasis vs EADV dermatitis | PDE4-modulator | Apremilast |
| Can be used vs should not be used | EADV dermatitis vs EuroGuiDerm dermatitis | Immunomodulator | Ciclosporin  Azathioprine |
|  | EADV dermatitis vs EuroGuiDerm psoriasis | Immunomodulator | Ciclosporin |
|  | EADV dermatitis vs AAD | Immunomodulator | Ciclosporin  Azathioprine |
|  | AAD vs EADV HS | TNF-inhibitor | Adalimumab  Infliximab |
| **PATERNAL USE** | | | |
| Rheumatology | | | |
| Insufficient data vs can be used | EULAR vs BSR | JAK-inhibitor | Tofacitinib  Filgotinib  Baricitinib  Upadacitinib |
|  |  | Interleukin inhibitor | Guselkumab  Risankizumab |
|  | ACR vs EULAR | Interleukin inhibitor | Ustekinumab  Tocilizumab  Secukinumab |
|  |  | Monoclonal antibodies | Belimumab  Abatacept |
|  | ACR vs BSR | JAK inhibitor | Tofacitinib  Baricitinib |
|  |  | Interleuking inhibitor | Ustekinumab  Tocilizumab  Secukinumab |
|  |  | Monoclonal antibodies | Belimumab  Abatacept |
| Dermatology | | | |
| Insufficient data vs should not be used | EuroGuiDerm psoriasis vs AAD | TNF-inhibitor | Adalimumab  Certolizumab pegol  Infliximab  Etanercept |
|  |  | Interleukin inhibitor | Ustekinumab  Secukinumab  Ixekizumab  Brodalumab |
|  | EuroGuiDerm psoriasis vs EuroGuiDerm dermatitis | Immunomodulator | Ciclosporin |
|  | EuroGuiDerm psoriasis vs EADV dermatitis | Immunomodulator | Ciclosporin |
|  |  | PDE4-modulator | Apremilast |
|  | AAD vs EuroGuiDerm dermatitis | Immunomodulator | Ciclosporin |
|  | AAD vs EADV dermatitis | Immunomodulator | Ciclosporin |
| Can be used vs should not be used | EuroGuiDerm psoriasis vs AAD | Immunomodulator | Methotroxate |
|  | EuroGuiDerm psoriasis vs EuroGuiDerm dermatitis | Immunomodulator | Methotrexate |
|  | EuroGuiDerm psoriasis vs EADV dermatitis | Immunomodulator | Methotrexate |
|  | EuroGuiDerm dermatitis vs AAD | Immunomodulator | Azathioprine |
|  | EADV dermatitis vs AAD | Immunomodulator | Azathioprine |

**Supplementary table 2** presents an overview of consistency in clinical guideline recommendations across gastroenterology, rheumatology and dermatology for pregnancy, lactation and paternal exposure. Consistency reflects the percentage of molecule-specific recommendations aligned between guidelines with available data. Totals are based on all guideline comparisons and may count medications multiple times if they appear in multiple guidelines. The PIANO, AGA and EADV guideline on hidradenitis suppurativa did not address paternal medication use. **Abbreviations**: ECCO (European, gastroenterology), PIANO (global consensus, gastroenterology), AGA (American, gastroenterology), EULAR (European, rheumatology), BSR (British, rheumatology), ACR (American, rheumatology), EuroGuiDerm dermatitis (European, dermatology), EADV dermatitis (European, dermatology), EuroGuiDerm psoriasis (European, dermatology), AAD (American, dermatology) EADV HS (European, dermatology

| Supplementary Table 2. Consistency of Guideline Recommendations Across Specialties (%) | | | |
| --- | --- | --- | --- |
| **Guideline comparisons** | **Pregnancy** | **Lactation** | **Paternal** |
| Gastroenterology vs Rheumatology | | | |
| ECCO vs EULAR | 12/14 (85.7) | 10/14 (71.4) | 8/9 (88.9) |
| ECCO vs BSR | 12/12 (100) | 8/13 (61.5) | 7/8 (87.5) |
| ECCO vs ACR | 11/13 (84.6) | 9/12 (75.0) | 8/9 (88.9) |
| PIANO vs EULAR | 10/15 (66.7) | 8/11 (72.3) | - |
| PIANO vs BSR | 15/15 (100) | 12/12 (100) | - |
| PIANO vs ACR | 10/12 (83.3) | 7/8 (87.5) | - |
| AGA vs EULAR | 9/10 (90.0) | 11/11 (100) | - |
| AGA vs BSR | 7/8 (87.5) | 8/10 (80.0) | - |
| AGA vs ACR | 8/10 (80.0) | 9/10 (90.0) | - |
| **Total** | **94/109 (86.2)** | **82/101 (81.2)** | **23/26 (88.5)** |
| Gastroenterology vs Dermatology (dermatitis) | | | |
| ECCO vs EuroGuiDerm | 3/3 (100) | 1/3 (33.3) | 1/2 (50.0) |
| ECCO vs EADV | 3/3 (100) | 2/3 (66.7) | 1/2 (50.0) |
| PIANO vs EuroGuiDerm | 3/3 (100) | 2/2 (100) | - |
| PIANO vs EADV | 2/2 (100) | - | - |
| AGA vs EuroGuiDerm | 1/2 (50.0) | 0/3 (0) | - |
| AGA vs EADV | 1/2 (50.0) | 2/3 (66.7) | - |
| **Total** | **13/15 (86.7)** | **7/21 (33.3)** | **2/4 (50.0)** |
| Gastroenterology vs Dermatology (psoriasis) | | | |
| ECCO vs EuroGuiDerm | 3/7 (42.9) | 2/4 (50.0) | 1/5 (20.0) |
| ECCO vs AAD | 8/12 (66.7) | 7/11 (63.6) | 5/8 (62.5) |
| PIANO vs EuroGuiDerm | 2/9 (22.2) | 1/2 (50.0) | - |
| PIANO vs AAD | 6/13 (46.2) | 4/8 (50.0) | - |
| AGA vs EuroGuiDerm | 2/6 (33.3) | 1/3 (33.3) | - |
| AGA vs AAD | 5/8 (62.5) | 4/9 (44.4) | - |
| **Total** | **26/55 (47.3)** | **19/37 (51.4)** | **6/13 (46.2)** |
| Gastroenterology vs Dermatology (Hidradenitis suppurativa) | | | |
| ECCO vs EADV HS | 0/2 (0) | 0/2 (0) | - |
| PIANO vs EADV HS | 0/2 (0) | 0/2 (0) | - |
| AGA vs EADV HS | 0/2 (0) | 0/2 (0) | - |
| **Total** | **0/6 (0)** | **0/6 (0)** | **-** |
| Rheumatology vs Dermatology (dermatitis) | | | |
| EULAR vs EuroGuiDerm | 4/6 (66.7) | 0/5 (0) | 2/7 (28.6) |
| EULAR vs EADV | 4/5 (80.0) | 2/5 (40.0) | 2/5 (40.0) |
| BSR vs EuroGuiDerm | 6/6 (100) | 4/6 (66.7) | 8/9 (88.9) |
| BSR vs EADV | 3/3 (100) | 4/4 (100) | 2/4 (50.0) |
| ACR vs EuroGuiDerm | 4/5 (80.0) | 1/3 (33.3) | 1/4 (25.0) |
| ACR vs EADV | 4/5 (80.0) | 3/4 (75.0) | 1/4 (25.0) |
| **Total** | **25/30 (83.3)** | **14/27 (51.9)** | **16/33 (48.5)** |
| Rheumatology vs Dermatology (psoriasis) | | | |
| EULAR vs EuroGuiDerm | 5/12 (41.7) | 2/4 (50.0) | 4/12 (33.3) |
| EULAR vs AAD | 12/18 (66.7) | 5/16 (31.3) | 8/14 (57.1) |
| BSR vs EuroGuiDerm | 2/14 (14.3) | 2/5 (40.0) | 1/14 (7.1) |
| BSR vs AAD | 9/18 (50.0) | 8/18 (44.4) | 9/15 (60.0) |
| ACR vs EuroGuiDerm | 3/9 (33.3) | 3/6 (50.0) | 4/8 (50.0) |
| ACR vs AAD | 11/15 (73.3) | 8/12 (66.7) | 5/11 (45.5) |
| **Total** | **42/86 (48.8)** | **28/61 (45.9)** | **31/74 (41.9)** |
| Rheumatology vs Dermatitis (Hidradenitis suppurativa) | | | |
| EULAR vs EADV HS | 0/2 (0) | 0/2 (0) | - |
| BSR vs EADV HS | 0/2 (0) | 0/2 (0) | - |
| ACR vs EADV HS | 0/2 (0) | 0/2 (0) | - |
| **Total** | **0/6(0)** | **0/6(0)** | **-** |

.

**Supplementary Table 3** provides a detailed overview of the observed inconsistencies across field comparisons, specifying the guidelines involved, the drug class and (if available) the molecule. If only class-level data were available, ‘NA’ was listed in the column ‘Molecule’. The guideline listed first in the table corresponds to the first-mentioned category in the table tittle (e.g., ‘insufficient data vs should not be used’ and ‘ECCO vs BSR’ indicates that ECCO recommended ‘insufficient data’ and BSR stated ‘should not be used’). To note: it is important to consider that inconsistencies between ‘insufficient data’ and ‘should not be used’ may only reflect discrepancies in phrasing rather than differing evidence bases. Some guidelines explicitly state that safety data are limited, while others recommend against the use of medications without clarifying whether this recommendation is driven by limited safety evidence or confirmed harm. **Abbreviations**: ECCO (European, gastroenterology), PIANO (global consensus, gastroenterology), AGA (American, gastroenterology), EULAR (European, rheumatology), BSR (British, rheumatology), ACR (American, rheumatology), EuroGuiDerm dermatitis (European, dermatology), EADV dermatitis (European, dermatology), EuroGuiDerm psoriasis (European, dermatology), AAD (American, dermatology) EADV HS (European, dermatology).

| Supplementary Table 3. Qualitative overview of the inconsistencies across specialties | | | | | | | | | | |
| --- | --- | --- | --- | --- | --- | --- | --- | --- | --- | --- |
| **Insufficient data vs should not be used** | | | | | | | | | | |
| Compared guidelines | Drug class | | Molecule | | | Compared guidelines | | Drug class | Molecule | |
| **PREGNANCY** | | | | | | **LACTATION** | | | | |
|  | | | | | | ECCO vs BSR | | JAK-inhibitor | Tofacitinib  Filgotinib | |
|  |  |  |  |  |  | ECCO vs EuroGuiDerm dermatitis | | Immunomodulator | Ciclosporin | |
|  |  |  |  |  |  | ECCO vs EuroGuiDerm psoriasis | | Immunomodulator | Ciclosporin | |
|  |  |  |  |  |  | ECCO vs AAD | | Immunomodulator | Ciclosporin  Tacrolimus | |
| AGA vs BSR | JAK-inhibitor | | Tofacitinib | | | AGA vs BSR | | Immunomodulator | Methotrexate | |
|  |  |  |  |  |  |  |  | JAK-inhibitor | Tofactinib | |
|  | | | | | | AGA vs ACR | | Immunomodulator | Methotrexate | |
|  |  |  |  |  |  | AGA vs EuroGuiDerm dermatitis | | Immunomodulator | Methotrexate | |
|  |  |  |  |  |  | AGA vs EADV dermatitis | | Immunomodulator | Methotrexate | |
|  |  |  |  |  |  | AGA vs EuroGuiDerm psoriasis | | Immunomodulator | Methotrexate | |
|  |  |  |  |  |  | AGA vs AAD | | Immunomodulator | Methotrexate | |
| EULAR vs ECCO | JAK-inhibitor | | Tofacitinib  Filgotinib | | | EULAR vs ECCO | | Immunomodulator | Methotrexate | |
| EULAR vs PIANO | JAK-inhibitor | | Tofacitinib  Filgotinib  Upadacitinib | | | EULAR vs PIANO | | JAK-inhibitor | Tofacitinib  Filgotinib  Upadacitinib | |
| EULAR vs EuroGuiDerm dermatitis | JAK-inhibitor | | Baricitinib  Upadacitinib | | | EULAR vs EuroGuiDerm dermatitis | | Immunomodulator | Methotrexate | |
|  |  |  |  |  |  |  |  | JAK-inhibitor | Baricitinib  Upadacitinib | |
| EULAR vs EADV dermatitis | PDE4-modulator | | Apremilast | | | EULAR vs EADV dermatitis | | Immunomodulator | Methotrexate  Mycophenolate mofetil | |
|  |  |  |  |  |  |  |  | PDE4-modulator | Apremilast | |
|  |  |  |  |  |  |  |  | PDE4-modulator | Apremilast | |
| EULAR vs EuroGuiDerm psoriasis | PDE4-modulator | | Apremilast | | | EULAR vs EuroGuiDerm psoriasis | | Immunomodulator | Methotrexate | |
| EULAR vs AAD | Immunomodulator | | Leflunomide | | | EULAR vs AAD | | Immunomodulator | Methotrexate  Leflunomide  Mycophenolate mofetil | |
| ACR vs ECCO | JAK-inhibitor | | Tofacitinib | | |  | | | | |
| ACR vs PIANO | JAK-inhibitor | | Tofacitinib | | | ACR vs PIANO | | JAK-inhibitor | Tofacitinib | |
| ACR vs EuroGuiDerm dermatitis | JAK-inhibitor | | Baricitinib | | | ACR vs EuroGuiDerm dermatitis | | JAK-inhibitor | Baricitinib | |
| ACR vs EADV dermatitis | PDE4-modulator | | Apremilast | | | ACR vs EADV dermatitis | | PDE4-modulator | Apremilast | |
| ACR vs EuroGuiDerm psoriasis | PDE4-modulator | | Apremilast | | |  | | | | |
| ACR vs AAD | Interleukin inhibitor | | Ustekinumab  Secukinumab | | |  |  |  |  |  |
| EuroGuiDerm psoriasis vs BSR | JAK-inhibitor | | Deucravacitinib | | |  |  |  |  |  |
| EuroGuiDerm psoriasis vs ACR | TNF-inhibitor | | Adalimumab  Infliximab  Etanercept | | |  |  |  |  |  |
| **Can be used vs should not be used** | | | | | | | | | | |
| Compared guidelines | Medication class | Molecule | | Compared guidelines | Medication class | | Molecule | Compared guidelines | Medication class | Molecule |
| **PREGNANCY** | | | | **LACTATION** | | | | **PATERNAL USE** | | |
|  | | | | ECCO vs EuroGuiDerm dermatitis | Immunomodulator | | Azathioprine | ECCO vs EuroGuiDerm dermatitis | Immunomodulator | Methotrexate |
|  |  |  |  |  | | | | ECCO vs EADV dermatitis | Immunomodulator | Methotrexate |
| ECCO vs EuroGuiDerm psoriasis | TNF-inhibitor | Adalimumab  Infliximab  Etanercept | |  |  |  |  |  | | |
| ECCO vs ACR | Interleukin inhibitor | Ustekinumab | |  |  |  |  |  |  |  |
| ECCO vs AAD | Immunomodulator | Azathioprine  Tacrolimus | | ECCO vs AAD | Immunomodulator | | Azathioprine  Thioguanine | ECCO vs AAD | Immunomodulator | Methotrexate  Azathioprine  Thioguanine |
| ECCO vs EADV HS | TNF-inhibitor | Adalimumab  Infliximab | | ECCO vs EADV HS | TNF-inhibitor | | Adalimumab  Infliximab |  | | |
| PIANO vs ACR | Interleukin inhibitor | Ustekinumab | |  | | | |  |  |  |
| PIANO vs EuroGuiDerm psoriasis | TNF-inhibitor | Adalimumab  Infliximab  Etanercept | |  |  |  |  |  |  |  |
| PIANO vs AAD | Immunomodulator | Azathioprine  Thioguanine | |  |  |  |  |  |  |  |
| PIANO vs EADV HS | TNF-inhibitor | Adalimumab  Infliximab | | PIANO vs EADV HS | TNF-inhibitor | | Adalimumab  Infliximab |  |  |  |
|  | | | | AGA vs EuroGuiDerm dermatitis | Immunomodulator | | Ciclosporin  Azathioprine |  |  |  |
| AGA vs EuroGuiDerm psoriasis | TNF-inhibitor | Adalimumab  Infliximab | | AGA vs EuroGuiDerm psoriasis | Immunomodulator | | Ciclosporin |  |  |  |
|  | | | | AGA vs AAD | Immunomodulator | | Ciclosporin Azathioprine  Thioguanine |  |  |  |
| AGA vs EADV HS | TNF-inhibitor | Adalimumab  Infliximab | | AGA vs EADV HS | TNF-inhibitor | | Adalimumab  Infliximab |  |  |  |
|  | | | |  | | | | EULAR vs ECCO | Aminosalicylates | Sulphasalazine |
| EULAR vs AGA | Immunomodulator | Ciclosporin | |  |  |  |  |  | | |
|  | | | | EULAR vs EuroGuiDerm dermatitis | Immunomodulator | | Ciclosporin  Azathioprine | EULAR vs EuroGuiDerm dermatitis | Immunomodulator | Methotrexate |
|  |  |  |  |  | | | | EULAR vs EADV dermatitis | Immunomodulator | Methotrexate  Mycophenolate mofetil |
| EULAR vs EuroGuiDerm psoriasis | TNF-inhibitor | Adalimumab  Infliximab  Etanercept | | EULAR vs EuroGuiDerm psoriasis | Immunomodulator | | Ciclosporin |  | | |
| EULAR vs AAD | Immunomodulator | Azathioprine | | EULAR vs AAD | Immunomodulator | | Ciclosporin  Tacrolimus  Azathioprine | EULAR vs AAD | Immunomodulator | Tacrolimus  Methotrexate  Azathioprine  Leflunomide  Mycophenolate mofetil |
| EULAR vs EADV HS | TNF-inhibitor | Adalimumab  Infliximab | | EULAR vs EADV HS | TNF-inhibitor | | Adalimumab  Infliximab |  | | |
|  | | | |  | | | | BSR vs ECCO | Aminosalicylates | Sulphasalazine |
|  |  |  |  | BSR vs EuroGuiDerm dermatitis | Immunomodulator | | Ciclosporin  Azathioprine | BSR vs EuroGuiDerm dermatitis | Immunomodulator | Methotrexate |
|  |  |  |  |  | | | | BSR vs EADV dermatitis | Immunomodulator | Methotrexate  Mycophenolate mofetil |
| BSR vs EuroGuiDerm psoriasis | TNF-inhibitor | Adalimumab  Infliximab  Etanercept | | BSR vs EuroGuiDerm psoriasis | Immunomodulator | | Ciclosporin |  | | |
| BSR vs AAD | Immunomodulator | Azathioprine | | BSR vs AAD | Immunomodulator | | Ciclosporin  Tacrolimus  Azathioprine | BSR vs AAD | Immunomodulator | Tacrolimus  Methotrexate  Azathioprine  Leflunomide  Mycophenolate mofetil |
| BSR vs EADV HS | TNF-inhibitor | Adalimumab  Infliximab | | BSR vs EADV HS | TNF-inhibitor | | Adalimumab  Infliximab |  | | |
|  | | | |  | | | | ACR vs ECCO | Aminosalicylates | Sulphasalazine |
| ACR vs AGA | Immunomodulator | Ciclosporin | |  |  |  |  |  | | |
|  | | | | ACR vs EuroGuiDerm dermatitis | Immunomodulator | | Ciclosporin | ACR vs EuroGuiDerm dermatitis | Immunomodulator | Methotrexate |
|  |  |  |  |  | | | | ACR vs EADV dermatitis | Immunomodulator | Methotrexate  Mycophenolate mofetil |
| ACR vs EuroGuiDerm psoriasis | TNF-inhibitor | Adalimumab  Infliximab  Etanercept | | ACR vs EuroGuiDerm psoriasis | Immunomodulator | | Ciclosporin |  | | |
| ACR vs AAD | Immunomodulator | Azathioprine | | ACR vs AAD | Immunomodulator | | Ciclosporin  Tacrolimus | ACR vs AAD | Immunomodulator | Methotrexate  Azathioprine  Leflunomide  Mycophenolate mofetil |
| ACR vs EADV hidradenitis suppurativa | TNF-inhibitor | Adalimumab  Infliximab | | ACR vs EADV HS | TNF-inhibitor | | Adalimumab  Infliximab |  | | |
| EuroGuiDerm dermatitis vs AGA | Immunomodulator | Ciclosporin | |  | | | |  |  |  |
| EADV dermatitis vs AGA | Immunomodulator | Ciclosporin | |  |  |  |  |  |  |  |
| EuroGuiDerm psoriasis vs AGA | Immunomodulator | Ciclosporin | |  |  |  |  |  |  |  |
| AAD vs PIANO | JAK-inhibitor | Tofacitinib | |  |  |  |  |  |  |  |
| AAD vs AGA | Immunomodulator | Ciclosporin | |  |  |  |  |  |  |  |
| AAD vs BSR | JAK-inhibitor | Tofacitinib | |  |  |  |  |  |  |  |
| AAD vs ECCO | JAK-inhibitor | Tofacitinib | |  |  |  |  |  |  |  |

**Supplementary Table 4** reflects the most frequently assigned recommendation per medication. The number of supporting guidelines is indicated between brackets.

| Supplementary Table 4. Cross-Guideline assigned Consensus on Medication Use in Reproductive Contexts | | | | |
| --- | --- | --- | --- | --- |
| **Drug class** | **Molecule** | **Pregnancy** | **Lactation** | **Paternal** |
| Conventional therapy | | | | |
| Aminosalicylates | Mesalazine | Can be used (3/3) | Can be used (3/3) | Can be used (1/1) |
|  | Sulphasalazine | Can be used (6/6) | Can be used (6/6) | Can be used (3/4) |
| Immunomodulators | Acitretin | Should not be used (2/2) | Should not be used (2/2) | Can be used (2/2) |
|  | Calcineurin inhibitor  Ciclosporin  Tacrolimus | Can be used (7/8)  Can be used (5/5) | Can be used (5/9)  Can be used (3/5) | Can be used (4/6)  Can be used (2/3) |
|  | Cyclophosphamide | Should not be used (1^st^ trimester) (2/2)  Can be used (2^nd^ and 3^rd^ trimester for severe maternal disease management) (2/2) | Should not be used (2/3) | Should not be used (3/3) |
|  | Hydroxychloroquine | Can be used (3/3) | Can be used (3/3) | Can be used (3/3) |
|  | Leflunomide | Should not be used (3/4) | Should not be used (3/4) | Can be used (3/4) |
|  | Methotrexate | Should not be used (10/10) | Should not be used (7/9) | Can be used (5/8) |
|  | Mycophenolate mofetil | Should not be used (6/6) | Should not be used (4/5) | Can be used (3/5) |
|  | Thiopurines  6-mercatopurine  Azathioprine  Thioguanine | Can be used (4/4)  Can be used (7/8)  Can be used (2/3) | Can be used (4/4)  Can be used (6/8)  Can be used (3/3) | Can be used (3/3)  Can be used (6/7)  Can be used (1/2)  Should not be used (1/2) |
| Biological therapy | | | | |
| Interleukin inhibitors | Anakinra | Can be used (2/3) | Can be used (3/3) | Can be used (3/3) |
|  | Bimekizumab | Can be used (1/2)  Insufficient data (1/2) | Can be used (1/1) | Can be used (1/2)  Insufficient data (1/2) |
|  | Brodalumab | Insufficient data (2/3) | Can be used (1/2)  Insufficient data (1/2) | Can be used (2/3) |
|  | Dupilumab | Insufficient data (2/2) | Insufficient data (2/2) | Insufficient data (2/2) |
|  | Guselkumab | Insufficient data (3/5) | Can be used (3/4) | Insufficient data (2/3) |
|  | Ixekizumab | Can be used (2/4)  Insufficient data (2/4) | Can be used (2/3) | Can be used (3/4) |
|  | Lebrikizumab | Insufficient data (1/1) | Insufficient data (1/1) | Insufficient data (1/1) |
|  | Mirikizumab | / | / | / |
|  | Risankizumab | Insufficient data (3/4) | Can be used (3/4) | Insufficient data (2/3) |
|  | Secukinumab | Can be used (2/5)  Insufficient data (2/5)  Should not be used (1/5) | Can be used (3/4) | Can be used (3/5) |
|  | Tildrakizumab | Can be used (2/4)  Insufficient data (2/4) | Can be used (2/3) | Can be used (1/2)  Insufficient data (1/2) |
|  | Tocilizumab | Can be used (2/3) | Can be used (3/3) | Can be used (2/4)  Insufficient data (2/4) |
|  | Tralokinumab | Insufficient data (1/1) | Insufficient data (1/1) | Insufficient data (1/1) |
|  | Ustekinumab | Can be used (5/8) | Can be used (5/7) | Can be used (3/5) |
| Other biological therapy | Abatacept | Can be used (2/3) | Can be used (3/3) | Can be used (2/4)  Insufficient data (2/4) |
|  | Belimumab | Can be used (2/3) | Can be used (3/3) | Can be used (2/4)  Insufficient data (2/4) |
|  | Rituximab | Can be used (3/3) | Can be used (3/3) | Can be used (4/4) |
|  | Vedolizumab | Can be used (3/3) | Can be used (2/3) | / |
| TNFα-inhibitors | Adalimumab | Can be used (7/9) | Can be used (7/8) | Can be used (5/6) |
|  | Certolizumab pegol | Can be used (8/8) | Can be used (8/8) | Can be used (5/6) |
|  | Etanercept | Can be used (6/7) | Can be used (5/5) | Can be used (5/6) |
|  | Golimumab | Can be used (7/7) | Can be used (7/7) | Can be used (5/5) |
|  | Infliximab | Can be used (7/9) | Can be used (7/8) | Can be used (5/6) |
| Small molecules | | | | |
| JAK-inhibitors | Abrocitinib | Should not be used (2/2) | Should not be used (2/2) | Can be used (2/2) |
|  | Baricitinib | Insufficient data (2/4)  Should not be used (2/4) | Insufficient data (2/4)  Should not be used (2/4) | Insufficient data (2/4)  Should not be used (2/4) |
|  | Deucravacitinib | Insufficient data (1/2)  Should not be used (1/2) | Should not be used (1/1) | Can be used (2/2) |
|  | Filgotinib | Should not be used (3/4) | Insufficient data (2/4)  Should not be used (2/4) | Can be used (2/3) |
|  | Tofacitinib | Can be used (1/7)  Insufficient data (3/7)  Should not be used (3/7) | Insufficient data (4/6) | Insufficient data (2/4)  Should not be used (2/4) |
|  | Upadacitinib | Should not be used (3/4) | Should not be used (3/4) | Can be used (2/3) |
| S1P-modulators | Etrasimod | Should not be used (1/1) | Should not be used (1/1) | / |
|  | Ozanimod | Should not be used (2/2) | Insufficient data (1/2)  Should not be used (1/2) | / |
| PDE4-modulator | Apremilast | Insufficient data (3/5) | Insufficient data (3/4) | Insufficient data (3/4) |
|  | Dimethyl fumarate | Should not be used (2/2) | Should not be used (2/2) | Can be used (1/1) |

**Supplementary Table 5** summarizes the methodological approaches applied in each guideline to develop and formulate clinical recommendations. **Abbreviations:** GRADE (Grading of Recommendation Assessment, Development and Evaluation)

| Supplementary Table 5. Methodological frameworks for developing recommendations across the included clinical guidelines | | |
| --- | --- | --- |
| **Guideline** | **Grading system** | **Base of recommendations** |
| Gastroenterology | | |
| 2025 PIANO Global Consensus | GRADE + RAND/UCLA Appropriateness Method | Evidence-based when sufficient evidence exists (GRADE), expert-derived appropriateness consensus (RAND/UCLA) when evidence is limited |
| 2022 ECCO | Oxford Centre for Evidence-based Medicine | Evidence level determined by study-design hierarchy |
| 2019 AGA | Consensus Clinical Care Pathway (no formal grading) | Multidisciplinary expert consensus based on available evidence |
| Rheumatology | | |
| 2024 EULAR | Oxford Centre for Evidence-based Medicine | Evidence level determined by study-design hierarchy, complemented by structured expert consensus voting |
| 2023 BSR | GRADE | Evidence-based when sufficient evidence exists (GRADE), with strength of recommendations (strong/weak) determined by benefit-risk balance |
| 2020 ACR | GRADE + consensus panel | Evidence-based when sufficient evidence exists (GRADE), combined with expert consensus to determine recommendation strength (strong/conditional) |
| Dermatology | | |
| 2025 EuroGuiDerm (psoriasis and dermatitis) | GRADE | Evidence-based when sufficient evidence exists (GRADE), using harmonized GRADE terminology for consistent recommendation phrasing |
| 2020 & 2019 AAD-NPF | Strength of Recommendation Taxonomy | Three-level evidence rating according to study design. Expert consensus applied when evidence was insufficient. |
| 2019 EADV (atopic dermatitis) | Consensus-based European guideline  (no formal grading) | Multidisciplinary expert consensus without a structured evidence-grading framework |
| 2015 EADV (hidradenitis suppurativa) | S1-level methodology  (European Dermatology Forum classification) | Expert consensus without formal or systemic evidence grading |
