# Supplementary material for: Inconsistent medication recommendations for immune-mediated inflammatory diseases across pregnancy, lactation, and paternal preconception: a guideline-based review
Source: Front Pharmacol. 2026 Feb 25;17:1744957. doi: 10.3389/fphar.2026.1744957 (PMC12975931; doi:10.3389/fphar.2026.1744957)
Supplement: Supplementary file 2 [file Image1.pdf]

Supplementary Figure 1 illustrates two examples of discrepancies between medication recommendations, each with a different underlying reason for the discrepancy.

Supplementary Figure 1. Examples of discrepancies in medication recommendations by underlying reason

Example 1: 'Insufficient safety data' vs 'should not be used'

Molecule (drug class): Tofacitinib (JAK inhibitors)
Guideline 1 (gastroenterology – AGA): Limited human data, consider other options → *No or insufficient safety information*
Guideline 2 (rheumatology – BSR): Not compatible with pregnancy (no explicit evidence of harm provided) → *Should not be used*

Discrepancy type: Different language, phrasing or formulation of recommendations

Example 2: 'Low risk' vs 'discontinue during pregnancy'

Molecule (drug class): Ustekinumab (interleukin inhibitor)
Guideline 1 (gastroenterology – ECCO): Low risk, limited data → *Can be used*
Guideline 2 (rheumatology – ACR): Conditionally recommend against, discontinue during pregnancy → *Should not be used*

Discrepancy type: Different recommendations on whether it should be used or not
